# Energy Efficient Routing Protocol in Sensor Networks Using Genetic Algorithm

**DOI:** 10.3390/s21217060

**Published:** 2021-10-25

**Authors:** Jatinkumar Patel, Hosam El-Ocla

**Affiliations:** Department of Computer Science, Lakehead University, Thunder Bay, ON P7B 5E1, Canada; jpatel12@lakeheadu.ca

**Keywords:** index terms—AODV, AOMDV, DSR, energy, genetic, optimization, performance, routing, sensors, network

## Abstract

In this paper, we examine routing protocols with the shortest path in sensor networks. In doing this, we propose a genetic algorithm (GA)-based Ad Hoc On-Demand Multipath Distance Vector routing protocol (GA-AOMDV). We utilize a fitness function that optimizes routes based on the energy consumption in their nodes. We compare this algorithm with other existing ad hoc routing protocols including LEACH-GA, GA-AODV, AODV, DSR, EPAR, EBAR_BFS. Results prove that our protocol enhances the network performance in terms of packet delivery ratio, throughput, round trip time and energy consumption. GA-AOMDV protocol achieves average gain that is 7 to 22% over other protocols. Therefore, our protocol extends the network lifetime for data communications.

## 1. Introduction

There are several types of wireless networks such as Mobile Ad-hoc Networks (MANETs) and wireless sensor networks (WSNs). In these networks, there is no infrastructure where the network is a set of multiple hop wireless nodes that communicate with other nodes without any centralized administrator. MANETs and mobile sensor networks permit self-organized connectivity [1] and each node communicates with other nodes directly or indirectly through intermediate nodes. In this regard, several issues should be considered such as multi-node routing, resources availability, and topology change. Therefore, a routing protocol in mobile networks must meet all these challenges to fulfill a high-performance requirement needed to achieve efficient data communication [2]. There are two main approaches for routing protocols: proactive and reactive protocols. In proactive protocols, mobile nodes must complete their routing tables by exchanging information about the network topology among the nodes. Proactive routing is also recognized by a table-driven routing because it manages routes to the final destination whether these routes are required or not [3]. Therefore, when there is a need for a route to a receiver, the path information is promptly available. In this proactive category, sending data will consume an obvious amount of energy while updated information is sent out unnecessarily when there is no data transmission. Accordingly, mobile nodes have inadequate power and their lifetime diminishes. On the other hand, in the reactive or on-demand routing approach, it has less routing overhead because it is not required a route management when there is no data transmission. In this case, routing data are reduced which in turn would minimize the traffic problems and cost.

Frequent node mobility is one of the challenges where the route may be broken and the end-to-end communication is terminated. In this case of node movement, the network will entirely get interrupted and consume more of the remaining energy. Additionally, having overload traffic requires an excessive amount of energy and also would diminish the network performance in terms of quality-of-service parameters. Reactive protocols consume less power; however, in case of route loss, these protocols start a new discovery process to search for an alternative route. Link failure also increases the load in the network and leads to a wastethe energy during the route discovery process. These all will result in a degradation of the network performance through increasing the load, reducing the throughput and hence the packet delivery ratio. Therefore, it is desired to develop a mechanism that uses less amount of energy and accordingly improves the performance. For efficient energy utilization at mobile nodes, the intensive study took place particularly to extend the lifetime of the network [4,5].

Ad-hoc on-demand multi-path distance vector (AOMDV) is a well-known routing protocol that selects routes based on the minimum number of hops. AOMDV provides alternative routes in case of nodes failure or channels disconnection and as a result, it is not needed to go through the route discovery phase and hence this would reduce the latency and maximize the throughput. On the contrary, if the route is broken for a node or a link defect in case of a single path routing approach, the data communication will be aborted and finding another route process should be triggered [6] and this, in turn, degrades the network performance. However, the AOMDV algorithm does not consider the availability of the node’s energy. In other words, a node might lose the power of its battery during a data communication which would consequently interrupt and this should switch to another available route. Routing algorithms based on saving energy were considered in separate studies where the cost was the main concern in [7] while in [8] the solution is complex and takes extended processing time.

In this paper, we propose an energy-efficient multi-path algorithm called genetic algorithm-based AOMDV (GA-AOMDV). Fitness function (FF) selects the route with nodes that maintains the least consumed energy utilizing routes selected by the AOMDV routing protocol. In other words, AOMDV returns multiple paths with the minimum number of hops. Next, FF is applied on the AOMDV routes to optimize the route going through nodes with the greatest residual energy. Our algorithm is a multi-path so, in case of nodes failure, alternative routes are available.

## 2. Related Work

There are various routing methods were proposed to enhance the network performance. The author in [9] developed a dynamic energy ad-hoc on-demand distance vector routing protocol (DE-AODV) to minimize the packet delay, maximize the network lifetime, and reduce energy consumption. The DE-AODV protocol is used to select the shortest route from the source to the destination with nodes that are energy-efficient. The early protocols proposed for proactive routing in ad hoc networks were destination sequenced distance vector (DSDV) [10] based on the Distributed Bellman-Ford (DBF) algorithm and the Optimized Link State Routing protocol (OLSR) [11]. In Link State schemes and when the network activity is low while the topology changes frequently, the amount of traffic can still be significant which would consume more energy. In [11], the authors introduced a routing mechanism where the residual energy together with the QoS metrics were considered. In this protocol, information about the network topology should be shared with the whole network. This would overwhelm the network with information that is not always necessary for all nodes and this, in turn, would degrade the performance of the network and lead to traffic problems such as data congestion especially in the case of dense networks. Another approach is the proactive path-finding algorithms. This approach combines features of the Distance Vector and Link State approaches and every node in the network constructs a Minimum Spanning Tree (MST). The Path Finding algorithm reduces both the amount of control data and also the possibility of temporary routing loops and this, in turn, would minimize the traffic problems. An example of this type of routing mechanism is the Wireless Routing Protocol (WRP) [12]. The main concern when applying the proactive approach to the WSN environment is the fact that as the network constantly changes, the cost of updating the topological information would be unduly high.

On the other hand, there are reactive routing protocols based on some type of request/reply scheme. A reactive mechanism seeks a process to find a route to the sink. Examples of reactive or on-demand protocols include the Ad hoc On-Demand Distance Vector (AODV) [13], the Dynamic Source Routing (DSR) [14] and the Temporally Ordered Routing Algorithm (TORA) [15]. In these reactive methods, the route discovery procedure is commonly used to find the best route. Mostly, shortest distance, the minimum number of hops, shortest latency, and least bandwidth usage may be used as the optimization conditions. These criteria are utilized to overcome main challenges in the network such as the low signal and attenuation, unreliable channels, higher delay, nodes mobility, and random packet loss.

In the case of nodes with high mobility speed, the network entirely might get interrupted and the remaining energy will be quickly depleted. In this case, AODV has to find another path as single-path reactive category protocols do. In TORA, route replies use controlled flooding to distribute the routing information through a form of a Directed Acyclic Graph (DAG) [16]. AODV and DSR protocols use unicast communication to direct the reply to the sender of the routing request. In AODV, the route information is stored at the next hop within the nodes on the path. The reversed path is engraved into the request packet as an accumulated route in the DSR and is used for source routing. AOMDV [17] is another reactive protocol based on AODV. AOMDV finds several alternative routes (multi-path) during the route discovery phase. As a result, AOMDV is an enhanced version of the AOMD where the network can adapt to one of the alternative paths during the failure of the current route and the transmission time is saved as no need to go often through the route discovery procedure for the established data communication session.

A genetic algorithm (GA) is a heuristic search algorithm based on natural selection and genetics. GA is an excellent approach to solving a problem for which little is known. Even if it is based on a random search, GA is able to provide a high-quality solution [18]. GA uses the principles of selection and evolution to produce multiple solutions to a given problem [19,20]. Challenges in wireless networks such as nodes mobility, fading, congestion, collision, etc. have no negative impact on the GA and this is one of its main characteristics. In [21], the authors proposed a GA-based routing algorithm for flying ad-hoc networks (FANETs). The proposed FF considers several parameters including the maximum link bandwidth, the highest network link stability, and the most residual power of the nodes. These parameters are more crucial in FANETs than in other types of ad-hoc networks. However, having several components in the fitness function will negatively impact the convergence speed of the algorithm to select the efficient route. Similarly, in [22] several fitness function components are proposed which will slow down the route selection process. In [23], the authors introduced a FF that is calculated using the shortest route length in addition to the parameters measured in the route discovery phase including the delay for getting the shortest route and the number of available routes. This method consumes an excessive amount of processing time and energy. In [24], authors proposed at FF that suits those applications that have an excessive amount of data transmission and therefore data congestion is a key that should be considered. There are other methods such as in [25] where the proposed FF is complex and the route selection takes an extended time. In [26,27,28], it was introduced GA-based methods which require lengthy processing and memory in the source node as every possible route would be involved in the GA regardless of its fitness level. In [29], the authors proposed a routing protocol that considers the network as a directed graph optimizing routes based on the quality of links between every two nodes using a genetic algorithm. In this regard, the link status might be impacted by several factors such as noise. Therefore, with a small change in a link status, the routing protocol should go through the regeneration of routes based on the modified graph.

Among the various maximum lifetime routing protocols, one type is called Efficient Power Aware Routing Protocol (EPAR) [30] which is the extension of the DSR protocol. EPAR selects the path that has the largest packet capacity at the smallest consumed packet transmission energy. Due to the mobility of the nodes in sensor networks, EPAR is proposed to find multi-path routes so the delay is taken to discover a new route will not consume time in case of the failure of the current route [17]. Alternatively, if a single intermediate node goes out of the coverage area, then the whole communication can be disturbed owing to topological change which would enlarge the data loss. This would result in an excessive amount of bandwidth usage and this, in turn, increases the number of packet retransmissions representing a low performance. Accordingly, EPAR using Breadth-First Search (BFS) was introduced to select the route with the minimum energy consumption. However, EPAR-BFS faces problems when the size of the network size increases.

In this paper, we propose a solution for selecting the energy-efficient route which enhances the mobile node’s lifetime.

## 3. Proposed Algorithm

### 3.1. Problem Statement

Most of the routing protocols focus on improving the performance of the sensors network in terms of the QoS at the expense of the routing processing time. Accordingly, the long processing time needed to select the best route would consume a considerable amount of energy. The power of sensors is mainly supplied by batteries which would be depleted with increasing the number of routing requests and their processing time that depends primarily on the routing algorithm complexity. Sensors would turn into being faulty nodes when their power is drained. Accordingly, this degrades the performance of the network regarding the data throughput and communication delay between the end nodes and hence alternative routes should be sought. To avoid having dead nodes particularly during the data communication period, routes should be selected to go through nodes with enough energy.

### 3.2. Proposed Method

AOMDV protocol returns routes based on the minimum number of intermediate nodes regardless of their power condition. To extend the lifespan of the nodes, we introduce a mechanism that utilizes the AOMDV array of routes and optimizes the one that has nodes with the maximum amount of residual energy. To do so, a fitness function (FF) is proposed to be applied on the AOMDV algorithm producing efficient routes with the minimum number of nodes with the least energy consumption. Next, we implement the GA which includes crossover and mutation phases where more potential routes are generated.

### 3.3. Fitness Function

In this section, we employ a fitness function that optimizes the route based on the maximum residual energy in its nodes. This function is defined as [31]:

*F_e_* = *E_x_/E_a_* (1)

where *F_e_* is the fitness function based on the energy. *E_x_* is the residual energy at each node *x* of one route and *E_a_* is the residual energy of all the nodes.

## 4. Methodology

**Figure 1:**
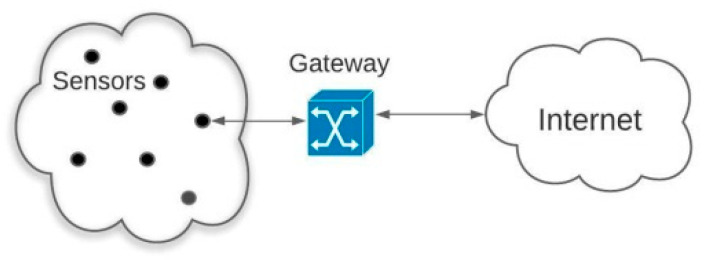
Wireless sensor network.

In Figure 1, WSN is shown where sensor nodes are connected wirelessly. Nodes may move randomly and this, in turn, would lead to channel breakage and topology change. Accordingly, another route should be discovered. We propose an algorithm that can provide an alternative efficient route if the primary one is disconnected.

Here, we explain the mechanism of our proposed GA-AOMDV method. This protocol generates a scores matrix representing a set of optimum routes between the source and the destination. AOMDV produces an array of alternative routes based on the minimum number of nodes. GA algorithm is applied on these routes devoted by AOMDV to generate more routes where the consumed energy at nodes is minimal.

In our algorithm, we assume all nodes have the same initial energy. GA has six steps explained below:

- Initialization: In this step, we set our assumptions for the needed parameters:
  ➢ Genes: This is the number of sensor nodes in a path before any data communication takes place,
  ➢ PopSize: This is the population size which is a set of all available routes between two end nodes,
  ➢ P_c_: This is the probability where a couple of routes may be crossed over,
  ➢ P_m_: This is the probability where a node in one route may be mutated,
  ➢ SurvivorSel: This is the survivor selection rule where a route can be considered as an accepted alternative based on its fitness score,
  ➢ GensNochange: This is the termination of the route search process where the array of available routes is sorted descendingly using the fitness values. At this stage, the optimized array has no further change.
- Fitness: We apply this function twice. Firstly, all routes returned by AOMDV are evaluated through the fitness function in Equation (1). Secondly, other routes generated through crossover and mutation phases are examined through this function to assess its efficiency as potential accepted route.
- Selection: Using the FF values, some of the AOMDV generated routes will be excluded to save the processing time needed for the next crossover and mutation. In doing this, the elitism method [32] is used where routes with small fitness scores will be removed from the selected routes pool. In other words, a route where its nodes have low energy will be excluded from the population of the parent routes where the crossover and mutation phases are applied. Therefore, the population will only represent elites which are the available routes with the minimum number of nodes and highest residual energy.
- Crossover: In this step, every couple of routes are paired and crossed over using the probability P_c_. Nodes are switched between each pair of routes with high fitness scores. The range of P_c_ is between 0.45 and 1 [32]. P_c_ is selected to be 0.5 in our simulation as it was indicated in [33] that it works perfectly with a large population size.
- Mutation: This phase is applied on the routes generated by AOMDV and crossover. In this step, the node order is altered in the same route using the probability P_m_. The range of P_m_ is between 0.001 and 0.5 [33]. In our simulation, P_m_ is selected to be 0.1 as it is commonly used by researchers [33]. New routes generated by crossover and mutation are assessed in the survivor select phase.
- Survivor selection: Each route generated by crossover and mutation phases is considered as a new child. If the fitness of the child is greater than the scores of its parents, then this child route will be added to the array of potential efficient routes; otherwise, this child route will be dropped. Optimized routes array is accordingly sorted and the route selected is the one with the highest fitness level. Other routes are alternatives that will be likely used whenever the selected route’s nodes become faulty or move out of the network area.

**Figure 2:**
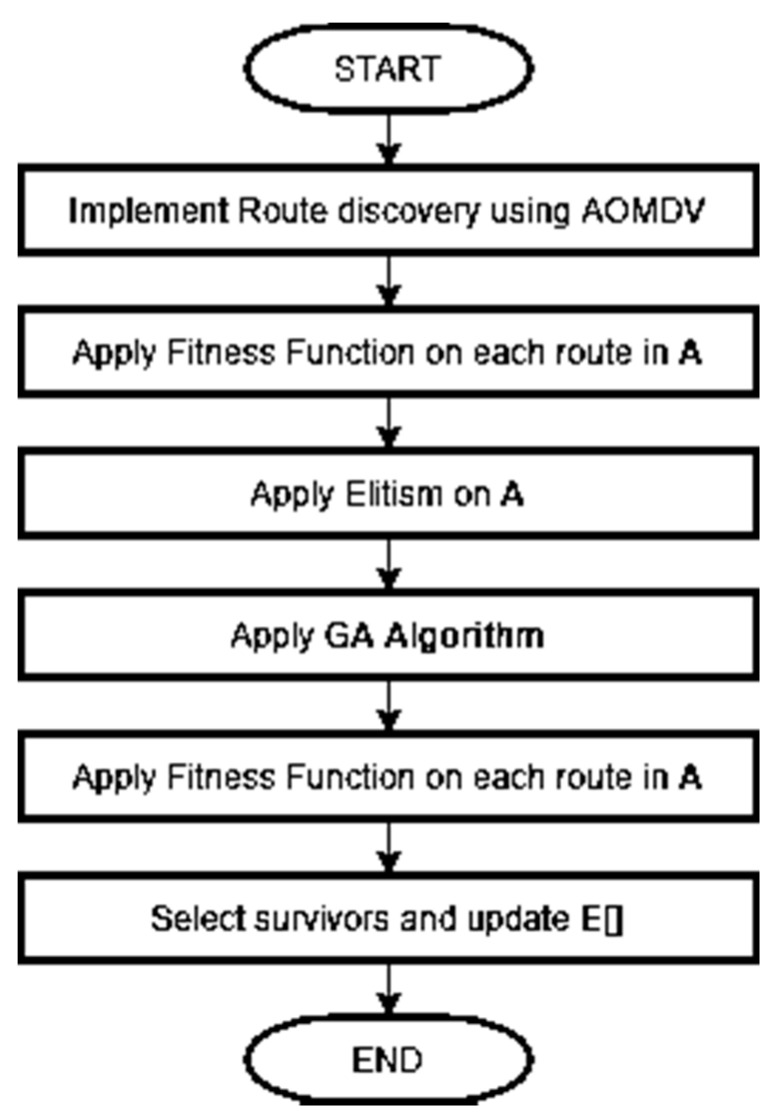
GA-AOMDV flowchart.

Figure 2 shows the steps that our routing protocol goes through to generate and select routes for data packets forwarding.

In Algorithm 1, E is the efficient routes array produced by the survivor selection process. The output routes generated by both crossover and mutation phases are C and M, respectively. POP_F is the population of the routes generated by AOMDV out of the PopSize where the elitism method is applied. FM, FP, and FO are the fitness scores F for the following: the child route generated by the mutation M process and the parents P and O generated by the AOMDV mechanism and/or crossover process. We summarize the steps of the GA-AOMDV algorithm as:

1. AOMDV protocol returns an array of routes (PopSize) based on the minimum number of hops (Genes). PopSize is the set of the parent routes where the GA will be applied to generate new child routes as explained below,
2. FF based on the minimum consumed energy of nodes involved in the PopSize is evaluated. Using the elitism method, routes with low fitness levels are dropped and only consider remaining routes as parents to be used in the GA,
3. Employ the crossover process over the elitism parent routes to generate new child routes through using P_c_.
4. Employ mutation process on the parent and child routes using P_m_,
5. Employ the FF again on every child route that can be considered as a potential route if its fitness score is higher than its parent routes fitness and this is the SurvivorSel in this step,
6. Store all the potential parent and child routes in an efficient routes array E,
7. Sort entries of array E in descending manner where the efficient route is the one with the highest fitness. Other routes in E will be utilized when the selected route fails because of channel disconnection or faulty nodes.

**Algorithm 1** Routing Protocol Algorithm1: **INPUT**: size of the network2: **OUTPUT**: Efficient array of routes3: Assumptions:4: Size: number of nodes5: Efficient_Paths: E []6: P_c_ = 0.57: P_m_ =0.18: Presnt_route = P, Old_route = O, New_route = N9: F(x) = Fitness Function (Min of energy consumption) for all nodes of each route x10: PopSize: Population of routes xs returned by AOMDV11: POP_F = Apply F(x) on routes of PoSize12: **while** (POP_F)13: Crossover (P, O, P_c_)14: Mutation (P, O, C, P_m_)15: **if** (FM ≥ FP)&(FM ≥ FO) **then**16: E [] = x17: **else**18: Drop x19: **end if**20: **end while**21: **return** routes array in E []

**Figure 3:**
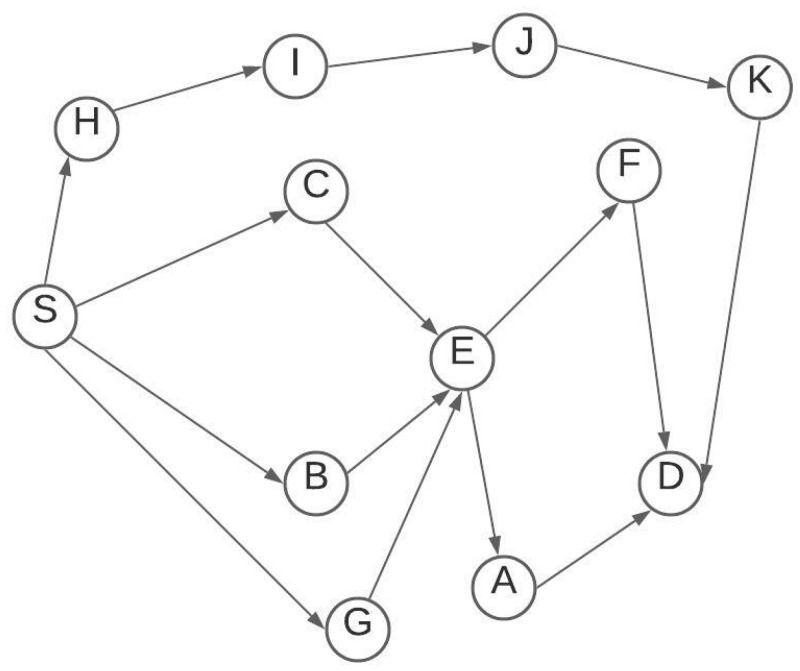
Network topology.

To better understand the algorithm, we consider the network shown in Figure 3 We assume the source node is S and the destination node is D. S will go through the route discovery phase using the AOMDV method. AOMDV will not select the route (S, H, I, J, K, D) because it does not have the least number of hops. AOMDV returns three routes including R1, R2, and R3 who have the minimum number of hops:

R1: S, C, E, F, D
R2: S, B, E, A, D
R3: S, G, E, A, D

We assume that the FF scores are 9.4, 9.1 and 4.7 for R1, R2, and R3, respectively. Using the elitism method, R3 will be dropped and only R1 and R2 will be considered for the GA application. Based on the crossover and mutation process, generated offspring are as follows:

R4: S, C, E, A, D
R5: S, B, E, F, D

The offspring will be checked by the FF evaluation where their scores are 9.6 and 9.8 for R4 and R5, respectively, so they will be added to the population. R5 has the highest score and this will be selected as the optimum route. If any failure occurred to R5 links or nodes during the data communication, R4 will replace R5.

## 5. Simulation, Parameters and Metrics

Our implementation is carried out using the NS2.35. It is a discrete-time event network simulator. In our simulation, we wrote an Object-Oriented Tool Command Language (OTcl) script to design the network in terms of its parameters and topologies such as traffic source, used routing algorithms, simulation time, the total number of nodes, sensors mobility speed and other parameters. NS2.35 provides a wide range of modules to employ in the simulation network such as different models of physical layer, different access control modules, transport layer protocols and applications [34]. Two files are produced when the simulation runs: Network AniMator (NAM) used to visualize the network and trace files which include a vestige of various events that happen during the simulation time. In Table 1, we assume the default network parameter values. We used a MAC based on IEEE 802.11 where Wireless Multimedia Sensor Networks (WMSNs) [35] and WSN [26] utilizing this standard in mesh networks are efficient solutions in various applications including security and ehealth [36] systems. The Xgraph program draws a graph on an X display given data read from either data files or from standard input if no files are specified. Xgraph in NS2 is used to plot the network parameter characteristics like throughput, delay, packet delivery ratio, latency, etc. We used Intel Dual-core 2.5 GHz with 2 GB RAM. In Table 1, we present our assumptions for the network configuration.

To evaluate the performance of routing protocols quantitative metrics are practiced [37]. We use the below performance metrics to evaluate our routing protocol.

(1) Throughput: This is the measure of how fast we can send packets through the network. The number of packets delivered to the receiver provides the throughput of the network. The throughput is defined as the total amount of data a receiver receives from the sender divided by the time it takes for the receiver to get the last packet. The throughput is calculated as:

(2) $$G=\frac{\sum B_{r}\times 8}{T}\times 10^{-6}\;\;\;\left[\mathrm{Mbps}\right]$$

where *G* is the throughput, *B_r_* is the total number of received bytes, and *T* is the simulation time.

(2) Energy Consumption: Battery energy consumption refers to the power spent in calculations that take place in the nodes for routing and other decisions. It is the power consumed in processing the packets per time unit. The energy is calculated as:

(3) $$E=\sum_{i=0}^{m}I_{i}-E_{i}\;\;\;\;\;\;\left[\mathrm{Joules}\right]$$

where *E* is the total energy consumption for all nodes *m*, *I_i_* is the initial energy at node *i* and *E_i_* is the energy at the end of the simulation process at node *i*.

(3) Packet Delivery Ratio: The ratio of the data packets delivered to the destinations to those generated by the constant bit rate (CBR) sources. It is the fraction of received packets by destination to the generated packets by source. Packet delivery ratio is calculated as:

(4) $$PDR=\frac{\sum N_{d}}{\sum N_{s}}\times 100\;\;\;\;\;\;\;\;\left[\%\right]$$

where *N_d_* represents the number of delivered packets and *N_s_* represents the number of sent packets.

(4) Round Trip-Time (RTT): This is the time measured from sending a route request packet (RREQ) until receiving a route reply packet (RREP). Having a long RTT indicates a slow data transmission and hence the throughput is low. To measure the RTT, we use a time stamp in the header option of the RREQ and RREP messages.

(5) Gain: To better understand the gain that GA-AOMDV can achieve compared to other protocols, including GA-AODV, LEACH-GA, EPAR, DSR, EPAR-BFS, AODV, we calculate the gain as:

(5) $$\mathrm{Gain}\backslash \mathrm{Saving}=\pm \left(\left(\sum y_{1i}-\sum y_{2i}\right)/\sum y_{2i}\right)\times 100\;\;\;\;\;\;\;\left[\%\right]$$

where

$y_{1i\;}$ is the performance value of the calculated metric for our protocol,
$y_{2i\;}$ is the performance value of the calculated metric for other protocols,
∑ is the summation over the range from *i* = 1 to the number of x-axis points of the metric y.

The metrics we evaluate are packet delivery ratio, energy consumption, throughput, and RTT. If Equation (5) produces a positive value, this indicates the gain that our method can achieve; otherwise, if the output is negative, it indicates the saving which would happen in the case of energy consumption and RTT.

(6) Average End-to-End delay: It denotes the average time required that a data packet delivers to the destination end.

(6) $$E2E=\sum_{i=1}^{n}\left(R_{i}-S_{i}\right)/n\;\;\;\;\;\;\;\;\;\;\left[\mathrm{Seconds}\right]$$

where

$R_{i}$ represents the simulation time in which the data packet *i*th delivered.
$S_{i}$ denotes the simulation time when the packet *i*th sent.
and *n* is the number of data packets delivered.

## 6. Results and Discussion

In this section, the results of the existing system and the proposed system are compared using the QoS parameters, including Packet Delivery Ratio, Average Throughput, RTT, and Average Energy Consumption with respect to the number of nodes. In the following, we provide results for two methods categories: methods based on GA and other methods not using GA.

### 6.1. Comparison with Genetic Algorithm-Based Protocols

This section compares our protocol with other genetic algorithm-based protocols, including LEACH-GA [28] and GA-AODV [31]. In this regard, GA-AODV is a single route topological reactive protocol that selects a route with the highest fitness among the discovered and generated routes. It keeps the record of the most optimized route for data forwarding and drops the other routes. Therefore, the protocol must go through the route discovery phase in the case of link failure, which imposes a significant load on the network. LEACH-GA is a clustering-based algorithm. This method uses GA to select the cluster head in each section.

**Figure 4:**
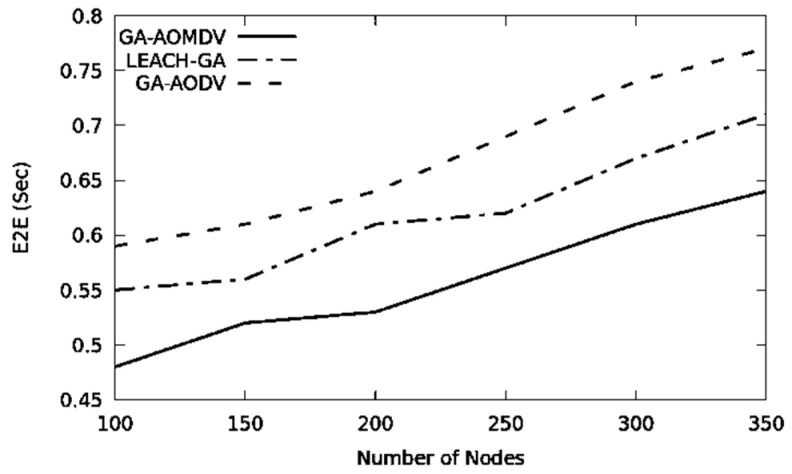
E2E vs. No of Nodes.

End-to-end delay is subjected to increase when the number of nodes in the network increases. Having a higher number of nodes increases the chance of having data congestion and this enlarges the queuing time. Additionally, the wireless channel is more occupied; therefore, a node in the network requires waiting for a longer period to access the medium and this extends the data transmission time. Selection of a route with the highest energy level can, therefore, maintain the load-balancing among nodes. GA-AOMDV is a multi-path routing protocol optimizing routes based on residual energy. Therefore, alternate routes and nodes are used whenever nodes fail. In all these cases, our mechanism will not go through the route discovery process and therefore the end-to-end delay would be minimal as shown in Figure 4.

**Figure 5:**
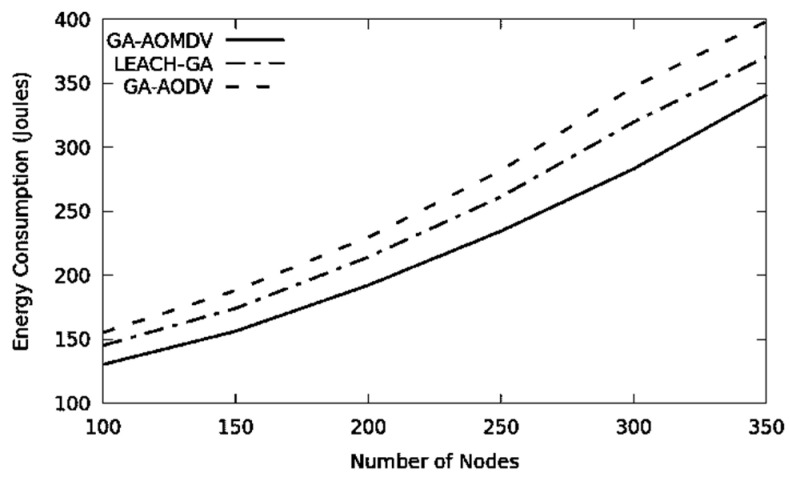
Energy consumption vs. No of Nodes.

When the network is congested where an excessive amount of routing packets circulates and more nodes are involved in the process of route finding and data transmission, obvious energy is consumed by network nodes. Frequent selection of cluster head process by LEACH-GA will consume energy and this does not happen with GA-AOMDV. On the other hand, GA-AODV is a single path routing mechanism and a new route is required every time a node failure occurs and therefore a route-finding process is triggered and thus consumes more energy. From all of that, GA-AOMDV has the least energy consumption as shown in Figure 5. Table 2 shows that GA-AOMDV saves energy with 11% to 19.65% compared to other protocols.

**Figure 6:**
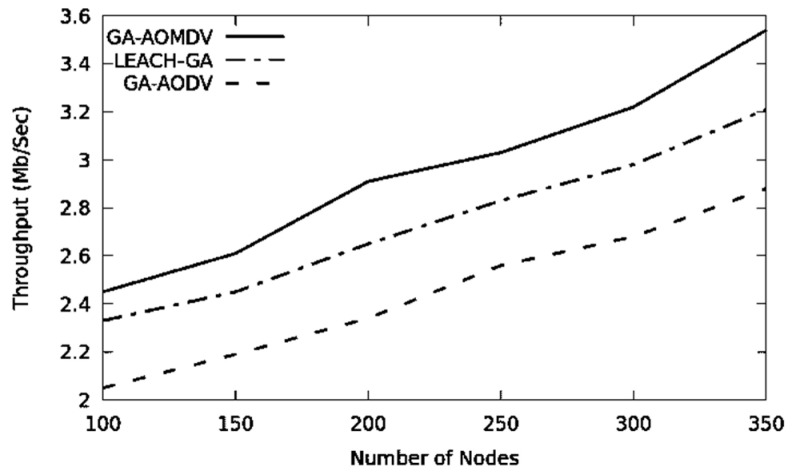
Throughput vs. No of Nodes.

**Figure 7:**
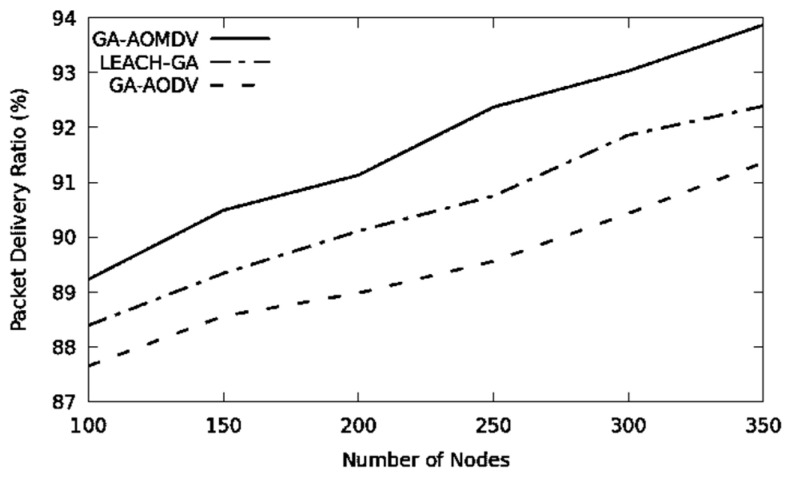
Packet delivery ratio vs. No of Nodes.

In Figure 6, expanding the number of nodes results in better connectivity and consequently a higher throughput. GA-AOMDV shows a superior performance due to alternative routes and the data load distributed more evenly through the network. Based on Table 3, GA-AOMDV improves throughput by 7.96% and 20.82% in comparison with LEACH-GA and GA-AODV, respectively. According to the given reasoning, the packet delivery ratio increases in Figure 7, where GA-AOMDV shows the highest performance followed by LEACH-GA and GA-AODV.

**Figure 8:**
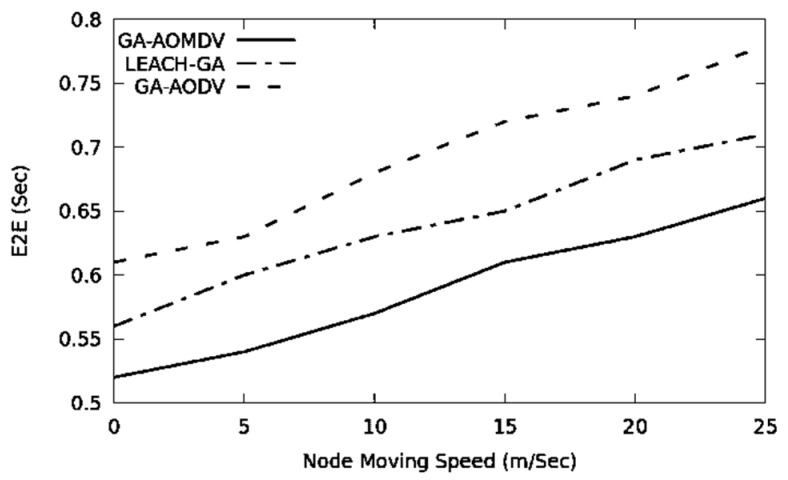
E2E vs. mobility speed.

The network topology is subjected to frequent change where there is nodal mobility. With a higher mobility speed, the topology changes more often; therefore, the performance degrades due to a higher chance of link failure. In Figure 8, the end-to-end delay increases with the higher mobility speeds. Every time a link fails to deliver data, the GA-AODV goes through the route discovery phase. Therefore, the data packet should remain in the queue link until a route is discovered. On the other hand, LEACH-GA has to go through cluster head selection more frequently. Therefore, data senders should adapt to the new cluster head, which increases the delay.

**Figure 9:**
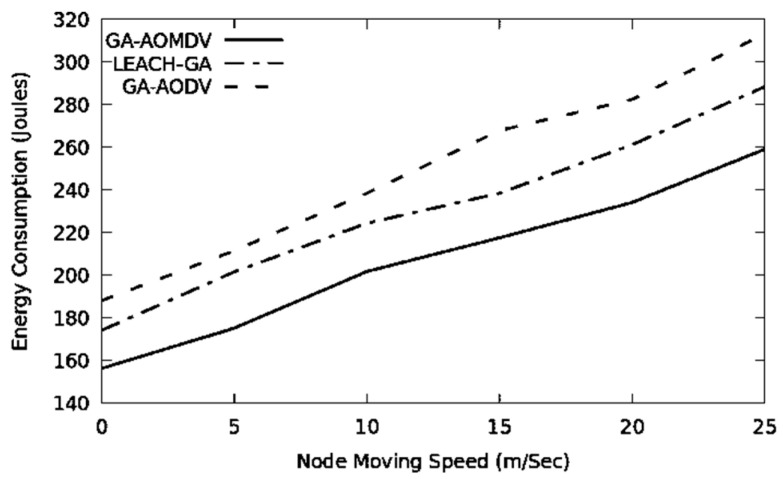
Energy consumption vs. mobility speed.

In Figure 9, GA-AODV needs to consume more energy as it is vulnerable to topological change and link failure. Additionally, LEACH-GA requires energy to adapt to the new topology. Having alternative routes helps GA-AOMDV to be more adaptable to the topological change. Therefore, it needs to produce less routing overhead and this reduces the energy consumption degradation.

**Figure 10:**
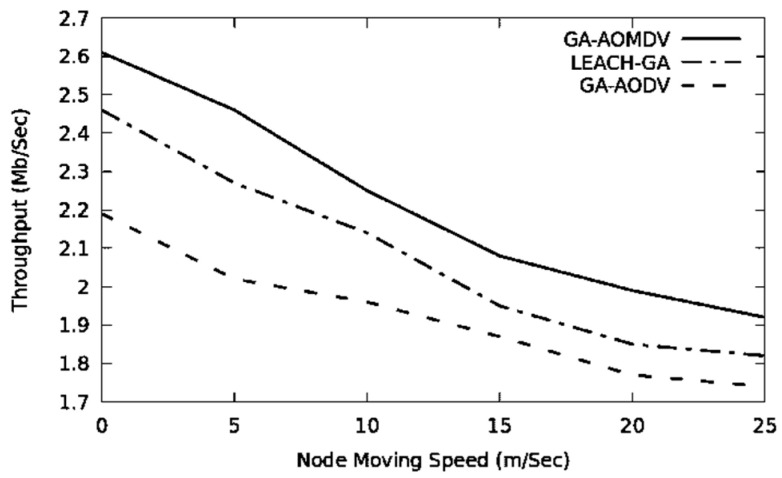
Throughput vs. mobility speed.

**Figure 11:**
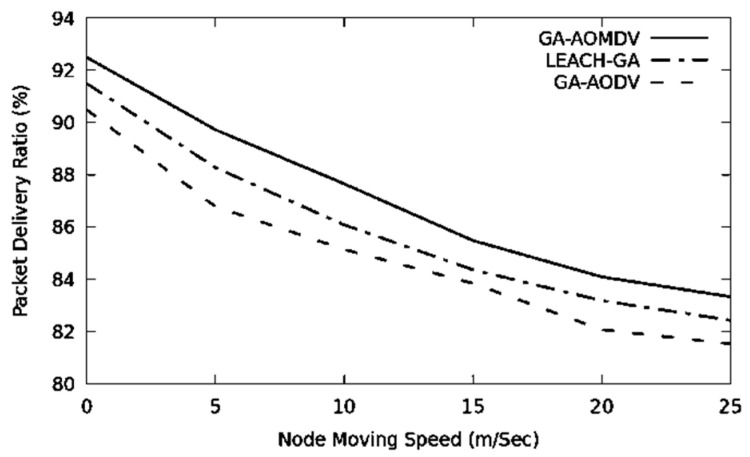
Packet delivery ratio vs. mobility speed.

The network topology changes more often when the mobility speed is higher. Therefore, the performance of the network drops in terms of throughput and packet delivery ratio, as it is depicted in Figure 10 and Figure 11. In this regard, GA-AOMDV shows adaptability where having more reliable routes are based on the least consumed energy at their nodes and also utilization of the GA mechanism.

### 6.2. Comparison with Non-Genetic Algorithm-Based Protocols

DSR protocol is based on the link-state algorithm in which each node would store the route to a destination. EPAR is a developed version of the DSR where the optimized route is the one that will consume less energy in the transmission of a data packet. EPAR-BFS proposes to enhance EPAR in the sense of selecting routes that pass through nodes with the minimum consumed energy. AODV is a single path protocol that selects the route with the minimum number of nodes and it is a hop-by-hop routing protocol. On the other hand, AOMDV is a multi-path routing protocol as an extended version of the AODV algorithm. As indicated earlier, the FF selects the routes that have the minimum consumed energy at the nodes. Therefore, GA-AOMD basically employs two criteria for the route selection: the smallest number of nodes (achieved by the AOMDV algorithm) and the least energy power used at the nodes (achieved by the GA algorithm). In this paper, it is worth comparing our GA-AOMDV with all these protocols to examine the performance of the network.

**Figure 12:**
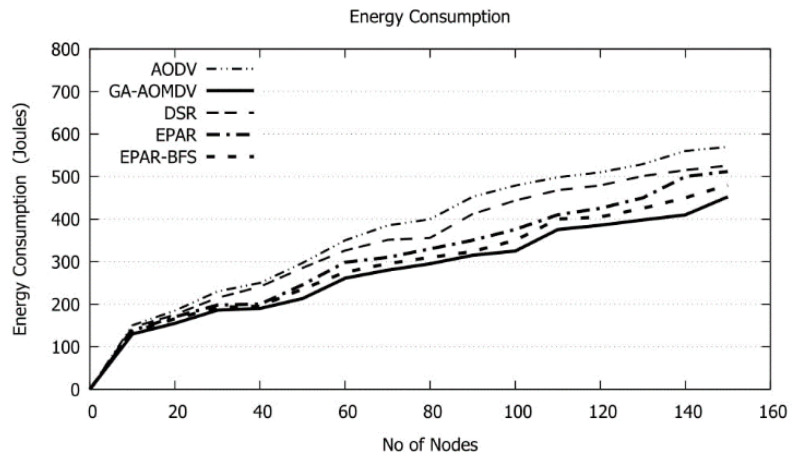
Energy Consumption vs. No of Nodes.

When the number of nodes increases beyond 40, the AOMDV route discovery phase in addition to the GA process will take a longer time resulting in producing additionally available routes. Moreover, when a channel disconnects or a node fails, triggering alternative route is processed. In all these cases, an excessive amount of energy is consumed as shown in Figure 12. However, GA-AOMDV has the minimum used energy as other efficient routes likely exist whenever are needed. We can observe that GA-AOMDV lessens the energy consumption with a range of 6% to 35% over other protocols. To better understand the saving in the energy consumption that GA-AOMDV can achieve compared to other protocols, we present in Table 4 data for results shown in Figure 12. Applying Equation (5) produces negative values which indicate the energy saving.

**Figure 13:**
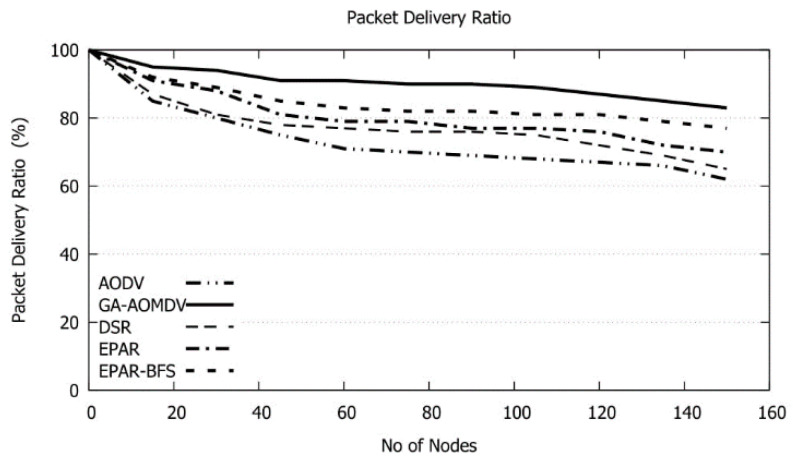
Packet Delivery Ratio vs. No of Nodes.

In Figure 13, GA-AOMDV outperforms other protocols. Our mechanism returns an array of energy-efficient routes so, in case of a link failure, alternative routes are always available without the need to go through the route discovery phase. When increasing the number of nodes particularly more than 40, more traffic problems such as congestion and data collision may arise and as a result, a lower amount of data is delivered to the final destination successfully. GA-AOMDV achieves a gain in packet delivery ratio with a range of 7% to 27% over other protocols. To better understand the gain in the packet delivery ratio that our method can achieve compared to other protocols, we present in Table 5 the data for results shown in Figure 13. Applying (5) produces positive values which indicate the gain in the packet delivery ratio obtained through using GA-AOMDV.

**Figure 14:**
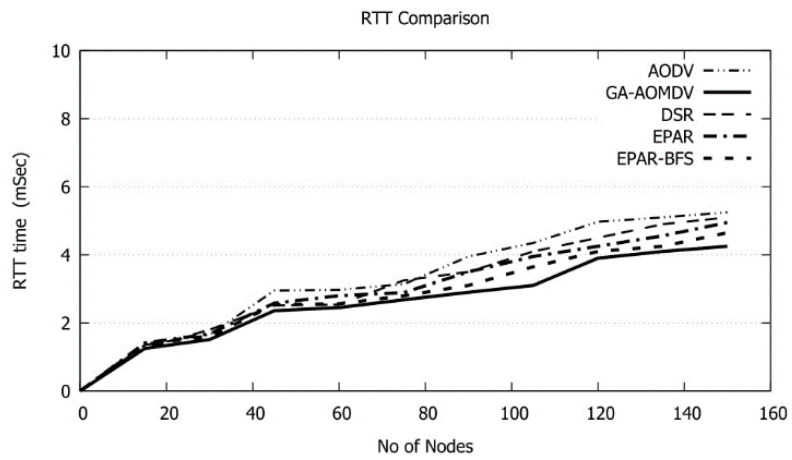
RTT vs. No of Nodes.

When the number of nodes increases, more data traffic issues arise. In this case, data packets retransmissions are invoked and accordingly RTT increases as shown in Figure 14. This in turn reduces the data delivery as shown in Figure 13; however, our protocol has the least RTT and this reflects the efficiency of the network performance utilizing our protocol. During the data transmission, a node may die because of losing the whole of its energy. In this case, the destination node will not receive the data packet and, therefore, the data acknowledgment will be delayed until the source node retransmits the data packet. This in turn will extend the RTT. Our protocol avoids such cases as the route will be selected based on the nodes with high residual energy. Therefore, the faulty node scenario will be avoided and hence the data packets likely arrive at the destination node promptly and RTT will be minimized. Reducing RTT is quite needed in data communications in FANETs which are sensitive to time in different military and civil applications. Particularly, small RTT is crucial in emergency messages transmission in vehicle ad hoc networks (VANETs) to alert vehicles to avoid accidents on the road.

**Figure 15:**
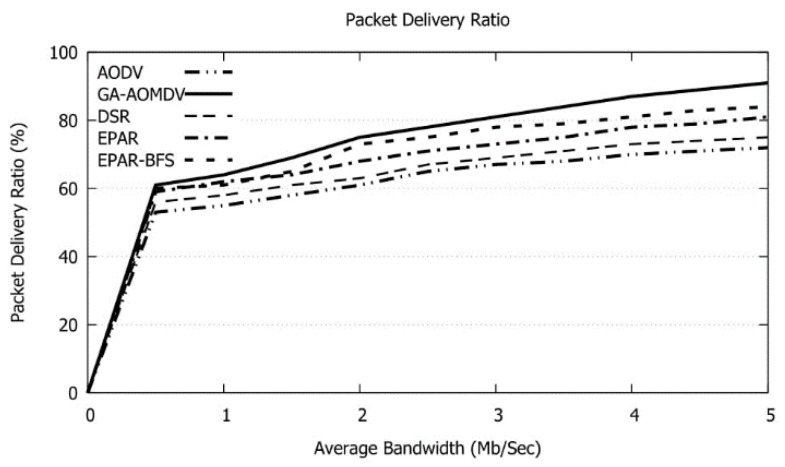
Packet Delivery Ratio vs. Average Bandwidth.

In Figure 15, when the bandwidth of the intermediate links increases, more data can be transmitted where our protocol achieves the best performance. GA-AOMDV gets a gain in packet delivery ratio with a range of 7% to 21% over other protocols.

**Figure 16:**
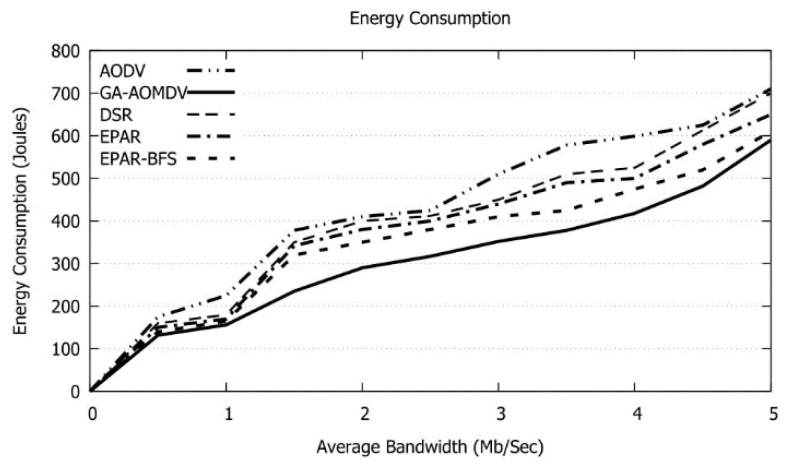
Energy Consumption vs. Average Bandwidth.

**Figure 17:**
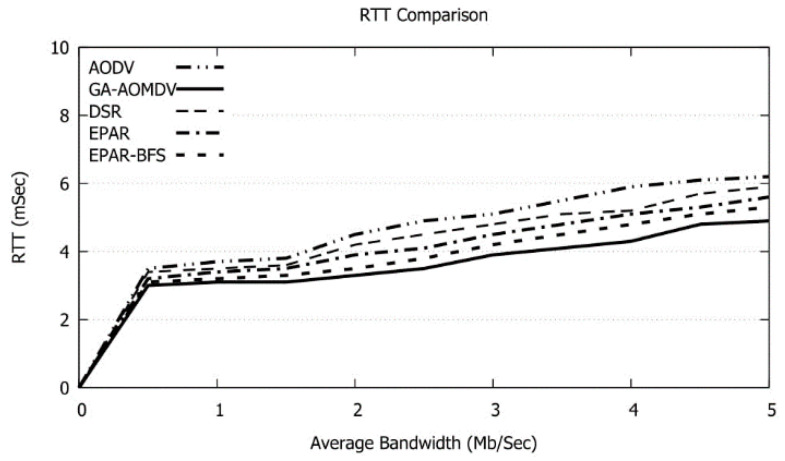
RTT vs. Bandwidth.

As increasing the bandwidth of the links, the amount of data transmission augments. As a result, the needed energy enlarges as shown in Figure 16. GA-AOMDV requires the minimum energy compared to other protocols. In addition, and as shown in Figure 17, RTT is obviously superfast with the bandwidth as explained above.

**Figure 18:**
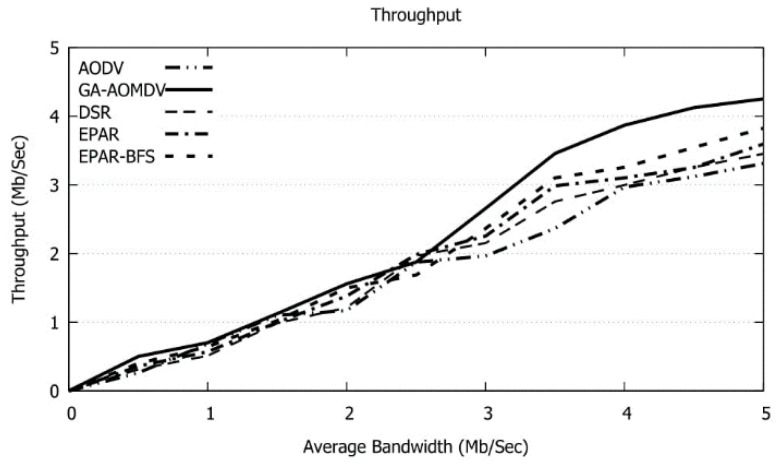
Throughput vs. Bandwidth.

In Figure 18 with a wider bandwidth range, more data packets can be transmitted. Our protocol selects nodes that have higher residual energy. Therefore, passing through nodes that might die particularly during the data communications, because of a lack of energy, will be avoided and hence this enhances the throughput. GA-AOMDV achieves a gain in throughput with a range of 9% to 20% over other protocols.

**Figure 19:**
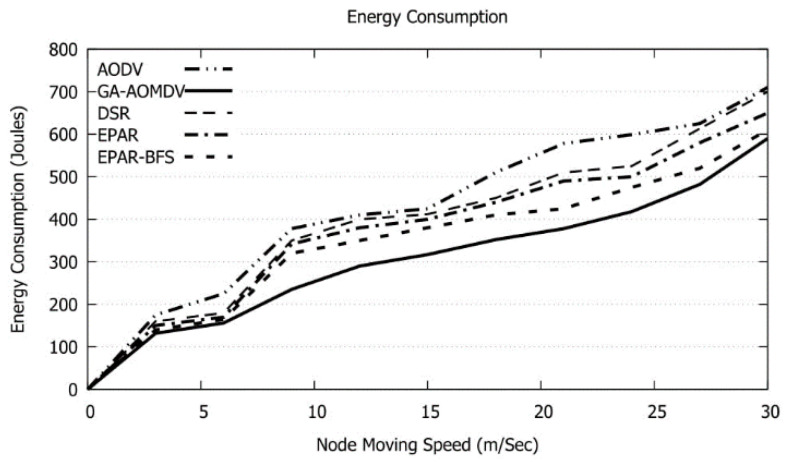
Energy Consumption vs. mobility speed.

**Figure 20:**
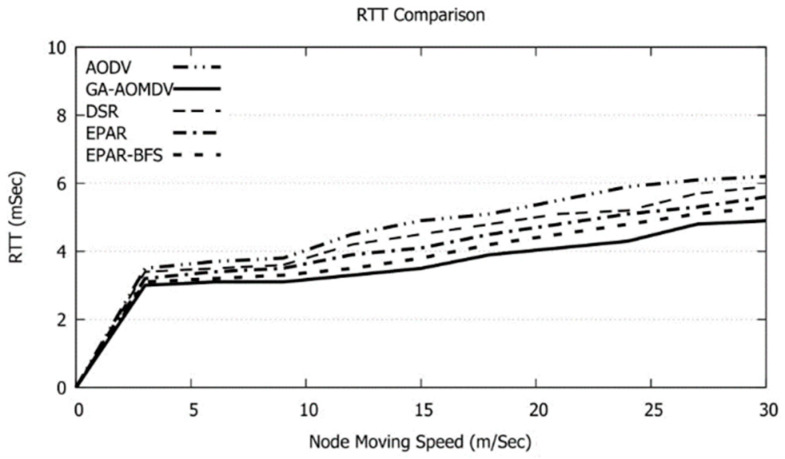
RTT vs. Speed of nodes.

Mobility of the nodes results in routes disconnection. As a result, alternative routes should be optimized through the routes discovery process and this, in turn, would maximize the energy consumption. GA-AOMDV avoids such a process as other possible routes likely already exist. Accordingly, the used energy will be minimized as shown in Figure 19. On the other hand, having mobile nodes will increase the possibility of traffic problems and this, in turn, requires packets retransmissions. In this case, RTT augments with the node speed as shown in Figure 20.

## 7. Protocols Analysis

WSN is composed of battery-driven nodes where computational power is quite limited in terms of memory and CPU processing capacity [38]. These resources are required to process the number of overhead packets which depend on the routing protocol complexity. Accordingly, these limited resources might be insufficient to process such time-consuming routing algorithms. For example, LEACH is a clustering-based mechanism that divides the network into clusters and selects a cluster head for each set of nodes and this, in turn, exploits a lot of energy particularly when the CPU has a low speed and hence spends an extended processing time. In addition, the cluster head might be quite far from the sink and this would use an extra amount of energy of nodes which could result in fast energy depletion. On the other hand, AODV and DSR are single path mechanisms that trigger the tight calculation of sensor resources for every route disconnect produced by link or node failure through the route discovery phase and this occurs frequently with the high mobility speed of sensors. This intensive route calculation also shortens the lifetime of nodes. In addition, DSR is a link-state protocol where all available routes in the network should be stored in the memory of each node. This would have another burden on the processing capacity which restricts the network scalability. Furthermore, EPAR, as a developed version of DSR, selects the routes based on the highest residual energy nodes; however, it is a single path scheme as well so it has the same problems as the DSR method. Therefore, we can understand that using such complex protocols will affect negatively the lifetime of nodes particularly with restricted processing resources even if the protocol selects the route based on the available energy as EPAR does. On the contrary, GA-AOMDV selects the routes based on the energy and also provides multipath routes and this, in turn, would save a lot of energy as shown in the results particularly with the high mobility speed of nodes. In addition, GA-AOMDV is a distance-vector mechanism that does not require a large memory as link-state-based protocols need.

## 8. Conclusions

In this paper, we propose a genetic algorithm (GA)-based Ad Hoc On-Demand Multipath Distance Vector routing protocol (GA-AOMDV). The fitness function is based on the energy consumption of nodes and therefore the AOMDV routes are optimized. As well-known, AOMDV selects the routes based on the minimum number of hops; therefore, GA-AOMDV applies two metrics to select the routes which are the least number of nodes in each route in addition to the minimum energy used. Before applying the GA, routes with low fitness scores are dropped and this accordingly reduces the processing time and speeds up the solution convergence. This is crucial in various applications bounded by time such as in FANETs and VANETs, particularly for alert messages communications. GA-AOMDV outperforms other protocols based on the QoS parameters. Particularly, our protocol reduces the energy consumption during data packets communications through mobile nodes. Therefore, the lifetime of the network is extended. Our protocol achieves an average gain of 7 to 22% over other protocols. As a result, it is obvious that the GA-based protocol enhances the performance of mobile networks. Moreover, GA-AOMDV does not require large processing resources as other protocols need.

## Figures and Tables

**Table 1:** Simulation Parameters.

Parameters	Values
Simulator	Network Simulator 2
Simulation Node	150
Interface Type	Phy/Wireless
Channel	Wireless Channel
Mac Type	MAC/IEEE 802.11
Queue Type	Queue/DropTail/Priqueue
Queue Length	201 packets
Antenna Type	Omni Antenna
Propagation Type	TwoRay Ground
Initial Energy	1000 J
Size of Packet	512 bytes
Protocol Algorithm	DSR, AODV, EPAR, GA_AOMDV, EPAR_BFS
Traffic	TCP/CBR

**Table 2:** Energy consumption values for Figure 5.

Energy (in Joules)/Protocol			
No of Node	GA-AOMDV	LEACH-GA	GA-AODV
100	130.22	145.11	155.23
150	156.26	174.13	188.08
200	192.20	214.18	229.64
250	234.49	261.32	281.73
300	283.11	319.63	347.16
350	341.05	370.62	398.30
Sum	1337.33	1484.99	1600.14
Saving %		11.04	19.65

**Table 3:** Throughput values for Figure 6.

Throughput (Mbps)/Protocol			
Node	GA-AOMDV	LEACH-GA	GA-AODV
100	2.45	2.33	2.05
150	2.61	2.45	2.19
200	2.91	2.65	2.34
250	3.03	2.83	2.56
300	3.22	2.98	2.68
350	3.54	3.21	2.88
Sum	17.76	16.45	14.7
Gain %		7.96	20.82

**Table 4:** Energy values of Figure 12.

Energy (in Joules)/Protocol					
No. of Nodes	AODV	GA-AOMDV	DSR	EPAR	EPAR-BFS
20.00	185.34	163.42	180.72	173.87	168.29
40.00	269.61	193.39	264.58	197.52	196.73
60.00	351.48	279.57	327.59	296.74	287.62
80.00	402.73	303.48	361.37	327.63	309.74
100.00	487.68	314.51	467.54	382.38	344.52
120.00	507.81	385.48	487.93	419.46	401.36
140.00	573.72	403.92	503.67	497.23	447.58
150.00	578.59	447.57	513.85	502.63	476.61
Sum	3356.96	2491.34	3107.25	2797.46	2632.45
Saving %	35		25	13	6

**Table 5:** Packet deliver ratio values of Figure 13.

	Packet Delivery Ratio (in %)/Protocol				
No. of Nodes	AODV	GA-AOMDV	DSR	EPAR	EPAR-BFS
20.00	82.12	96.35	83.59	88.24	90.83
40.00	78.34	92.48	80.13	84.75	88.29
60.00	73.75	91.35	78.52	80.51	84.11
80.00	68.41	90.83	76.68	78.25	82.75
100.00	67.28	89.21	75.32	76.48	81.85
120.00	66.82	85.65	71.78	74.35	80.27
140.00	63.56	83.28	68.71	70.13	79.42
150.00	61.34	82.54	65.29	69.39	78.69
Sum	561.62	711.69	600.02	622.10	666.21
Gain %	27		19	15	7
